# TGFβ1 Attenuates Expression of Prolactin and IGFBP-1 in Decidualized Endometrial Stromal Cells by Both SMAD-Dependent and SMAD-Independent Pathways

**DOI:** 10.1371/journal.pone.0012970

**Published:** 2010-09-24

**Authors:** Nicole M. Kane, Marius Jones, Jan J. Brosens, Rodney W. Kelly, Philippa T. K. Saunders, Hilary O. D. Critchley

**Affiliations:** 1 Medical Research Council Human Reproductive Sciences Unit, Centre for Reproductive Biology, Queen's Medical Research Institute, Edinburgh, Scotland, United Kingdom; 2 Institute of Reproductive and Developmental Biology, Imperial College School of Medicine, Hammersmith Hospital, London, United Kingdom; 3 Division of Reproductive and Developmental Sciences, Centre for Reproductive Biology, Queen's Medical Research Institute, The University of Edinburgh, Edinburgh, Scotland, United Kingdom; Cincinnati Children's Research Foundation, United States of America

## Abstract

**Background:**

Decidualization (differentiation) of the endometrial stromal cells during the secretory phase of the menstrual cycle is essential for successful implantation. Transforming Growth Factor β1 (TGFβ1) canonically propagates its actions via SMAD signalling. A role for TGFβ1 in decidualization remains to be established and published data concerning effects of TGFβ1 on markers of endometrial decidualization are inconsistent.

**Methodology/Principal Findings:**

Non-pregnant endometrial stromal cells (ESC) and first trimester decidual stromal cells (DSC) were cultured in the presence or absence of a decidualizing stimulus. Incubation of ESCs with TGFβ1 (10 ng/ml) down-regulated the expression of transcripts encoding the decidual marker proteins prolactin (PRL), insulin-like growth factor binding protein-1 (IGFBP-1) and tissue factor (TF). TGFβ1 also inhibited secretion of PRL and IGFBP-1 proteins by ESCs and surprisingly this response preceded down-regulation of their mRNAs. In contrast, DSCs were more refractory to the actions of TGFβ1, characterized by blunted and delayed down-regulation of PRL, IGFBP-1, and TF transcripts, which was not associated with a significant reduction in secretion of PRL or IGFBP-1 proteins. Addition of an antibody directed against TGFβ1 increased expression of IGFBP-1 mRNA in decidualised cells. Knockdown of SMAD 4 using siRNAs abrogated the effect of TGFβ1 on expression of PRL in ESCs but did not fully restore expression of IGFBP-1 mRNA and protein.

**Conclusions/Significance:**

TGFβ1 inhibits the expression and secretion of decidual marker proteins. The impact of TGFβ1 on PRL is SMAD-dependent but the impact on IGFBP1 is via an alternative mechanism. In early pregnancy, resistance of DSC to the impact of TGFβ1 may be important to ensure tissue homeostasis.

## Introduction

Decidualization, the process by which progesterone acts on the estrogen-primed endometrium to convert precursor stromal cells into decidual cells, is essential for successful implantation and maintenance of pregnancy (reviewed in [1], [2], [3]). The decidualization reaction is initiated in the perivascular stromal cells and under the influence of progesterone, spreads ‘wave-like’ throughout the stromal region. It is characterised by the phenotypic transformation of the elongated fibroblast-like endometrial stromal cells (ESC) into a larger, spherical decidual cell. This change in cell shape is associated with rearrangements in cellular architecture, the accumulation of glycogen and increased expression of prolactin (PRL) and insulin growth factor binding protein 1 (IGFBP-1) [4], [5], [6]. Studies using primary cultures of human endometrial stromal cells (ESCs) have revealed that this process is complex and likely to involve multiple factors including PGE_2_, relaxin and cAMP in addition to progesterone [4], [7], [8], [9].

A strong association exists between the degree of trophoblast invasion and the extent of decidualization in species with a hemochorial placenta [10]. Not only does human trophoblast exhibit the greatest degree of trophoblast invasion observed in all species, but also human endometrium undergoes the most extensive decidualization reaction [11], [12]. Decidualized stromal cells are temporally and spatially positioned to promote local homeostasis during implantation and counteract the threat of haemorrhage during trophoblast invasion [13]. Production of tissue factor by decidual cells is also thought to be important in preventing uterine bleeding in the peri-implantation phase of the cycle [14].

It has been hypothesised that menstruation only occurs in species where the decidualization reaction is initiated spontaneously during each cycle regardless of the presence of a blastocyst [10]. Although the classic “trigger” for the onset of menstruation is the withdrawal of progesterone associated with the demise of the corpus luteum, multiple cytokines and growth factors have been reported to play a role in this process [15], [16], [17]. Based on several lines of evidence we have previously proposed that transforming growth factor β1 (TGFβ1), which canonically transduces its signal from type II serine/threonine kinase transmembrane receptors to the nucleus through the Sma- and mothers against decapentaplegic (MAD)-related protein (SMAD) signalling cascade [18], might play a role in initiating the process of menstruation [19]. For example, expression of TGFβ1 is increased in stromal cells as they undergo decidualization [20], [21]. We have previously demonstrated that treatment of decidualized ESC with TGFβ1 suppresses expression of progesterone receptor (PR) suggesting that TGFβ1 may potentiate the effects of progesterone withdrawal[19]. In contrast, several studies have reported that TGFβ1 may increase expression of PRL in endometrial stromal cells (ESC) [21], [22].

An enhanced understanding of local mechanisms involved in the regulation of endometrial events preceding menstruation is an essential prerequisite for delineating the aetiology of early pregnancy complications as well as abnormal endometrial tissue activity associated with common gynaecological complaints such as heavy menstrual bleeding (HMB). In the current study we have complemented and extended our previous investigation [19] by investigating the possibility that local production of TGFβ1 within the endometrium plays a critical role in triggering the process of menstruation in cells from non-pregnant endometrium by inhibiting biosynthesis and/or secretion of PRL, IGFBP-1 and tissue factor (TF) via a SMAD-dependent pathway. We have also examined the effects of TGFβ1 in cells obtained from early pregnancy to compare the TGFβ1 response between stromal cells decidualized *in vitro* and *in vivo*.

## Materials and Methods

### Patients and tissue collection

Human endometrial tissue specimens (n = 20; proliferative and secretory phase samples) were obtained from women undergoing surgery for benign gynaecological conditions. Written informed patient consent was provided prior to tissue collection. Local research ethical committee approval for the study was granted. Biopsies were collected with an endometrial suction curette (Pipelle, Laboratoire CCD, Paris, France) or alternatively, full thickness endometrial samples were obtained. These latter biopsies included superficial and basal endometrium plus the endometrial-myometrial junction. All patients were of reproductive age, described regular menstrual cycles between 25–35 days and had not received exogenous hormones or used an intrauterine contraceptive device in the three months prior to surgery. All subjects had a serum sample collected at the time of surgery for the determination of circulating estradiol (E_2_) and progesterone (P) levels by Radio Immunoassay (RIA). All samples were consistent for the designated cycle stage based on standard histological criteria of Noyes et al [23], the patient's reported last menstrual period and circulating E_2_ and P levels at time of biopsy collection.

Decidual tissue specimens were obtained from women (n = 7; 8–10 weeks gestation) who had undergone surgical termination of pregnancy during the first trimester of pregnancy. All women had an ultrasound scan to confirm viability of pregnancy and gestational age. All material from the suction curettage procedure was collected. Decidua parietalis tissue (n = 7) was selected by macroscopic inspection from the products of the termination aspiration procedure and subjected to cytokeratin staining to confirm exclusion of trophoblast. Endometrial and decidual tissue was collected in sterile RPMI 1640 culture medium (Sigma, Poole, Dorset, UK) and processed in one of two ways: fixed in 10% neutral buffered formalin (NBF) 24 h at 4°C followed by storage in 70% ethanol prior to wax embedding, or used for isolation of primary stromal cells as detailed below.

### Isolation of stromal cells from non-pregnant endometrium

Endometrial specimens (n = 21) were separated into epithelial and stromal cell preparations by enzymatic digestion as previously described [19]. Briefly, specimens were washed in Dulbecco's Phosphate Buffered Saline (Sigma), minced into 1 mm^3^ pieces and digested in collagenase (1 mg/ml, Sigma) and DNAase (0.1 mg/ml, Sigma) for 80 min at 37°C. Repeated passage through an 18 g needle was used to aid tissue dispersion. The tissue homogenate was re-suspended in 10 ml of RPMI 1640 medium (Sigma) and centrifuged (1700 rpm, 3 min). Cell pellets were then re-suspended in 10 ml of RPMI 1640 medium (Sigma) supplemented with 10% fetal calf serum (FCS) (Mycoplex, PAA Laboratories, Kingston-Upon-Thames, UK), penicillin (50 µg/ml; Sigma), streptomycin (50 µg/ml; Sigma) and gentamycin (5 µg/ml; Sigma) and the stromal cells were separated from endometrial glands by filtration through a 73 µm nylon sieve (Falcon, VWR International Ltd, Leicestershire, UK). The filtrate, containing the primary ESC, was plated in 75 cm^3^ culture flasks (Corning Incorporated, Corning, NY) for a minimum period of 5 days and allowed to reach confluence.

### Isolation of stromal cells from 1st trimester deciduas

All decidual samples were processed to remove both glandular material and deplete them of CD56^bright^ uterine natural killer cells (uNKs). Briefly, decidual tissue was finely minced into 1 mm^3^ sections using surgical blades (Swann, Morton Ltd, Sheffield, UK) and residual blood clots were removed. Approximately 10 g of the minced tissue was placed in 20 ml of RPMI with 10% FCS, 4 ml of collagenase (2 mg/ml) and 0.5 ml of DNAse (0.1 mg/ml; Sigma) for 1 h 20 min on a roller at 37°C. After digestion, 30 ml of RPMI 10% FCS was added and the mixture was left to stand for 5 min to allow sedimentation. The supernatant was decanted by aspiration and passed sequentially through 73 µm and 40 µm filters (VWR). The filtrate was centrifuged at 400 g for 5 min and the resulting cell pellet was re-suspended in 15 ml of PBS supplemented with 2% FCS and 0.1% NaN_3_ and subsequently overlaid onto 15 ml of Lymphoprep™ (Axis-Shield, Oslo, Norway) before further centrifugation at 710 g for 20 min with no brake. The cells at the interface were collected; these consisted of 60–80% uNKs, 5–15% CD14^+^ macrophages, 10–20% T cells as well as stromal and epithelial cells. Cells were washed in 20 ml of RPMI 10% FCS and centrifuged at 710 g for 5 min. CD56^bright^ uNKs were removed by positive selection using CD56 antibody-coated magnetic Microbeads as previously described[24]. The remaining cells were transferred to a 75 cm^3^ cell culture flask in 10 ml of RPMI 10 FCS and incubated at 37°C in a humidified atmosphere of 5% CO_2_. After 24 h the media was changed non-adherent cells were discarded and the adherent decidualized stromal cells (DSCs) were allowed to attain confluence and used for experimentation at first passage.

### *In vitro* primary cell culture experiments

ESCs and DSCs were maintained at 37°C in 5% (v/v) CO_2_ in RPMI 1640 medium (Sigma) supplemented with 2% FCS (Mycoplex), penicillin (50 µg/ml; Sigma), streptomycin (50 µg/ml;Sigma) and gentamycin (5 µg/ml; Sigma). The cells were seeded in 6-well plates at a concentration of 2.5×10^5^ cells/ml and allowed to adhere and attain 90% confluence. Supernatant was changed every 3 days. Decidualization of the cells was induced in decidualization medium (DM) consisting of RPMI 1640 medium containing 2% FCS, 8-Bromoadenosine 3′,5′-cyclic monophosphate sodium salt (8-Br-cAMP) (0.5 mM; Sigma) and 6α-Methyl-17α-acetoxyprogesterone (MPA) (1 µM) for 6 days. Following decidualization cells were incubated in 2% FCS RPMI 1640 and DM containing TGFβ1 (R&D Systems, Abingdon, UK) for up to 72 h.

### Targeted knockdown of SMAD 4

Two HP GenomeWide siRNA duplexes to SMAD 4 (Genbank accession no. NM_005359) were purchased from Qiagen (Crawley, United Kingdom): SMAD 4-1 (5′- AAGCAGCGTCACTCTACCTAA), SMAD 4-2 (5′- CCCTGTTAAACAGTAGTTGTA). An additional siRNA (Qiagen), targeting MAPK (5′- AATGCTGACTCCAAAGCTCTG) and a non-silencing control (5′-AATTCTCCGAACGTGTCACGT) were used in all experiments (Qiagen). In addition, a negative control duplex (5′-AATTCTCCGAACGTGTCACGT) labelled with Alexa Fluor 488 (Qiagen) was used to monitor transfection efficiency. Cells were transfected with siRNA duplexes using HiPerfect transfection reagent (Qiagen). All experiments were performed in duplicate using cells in 6-well culture dishes at 70% confluence [19]. Cells were decidualized *in vitro* for 36 h, washed twice with PBS, transfected with duplexes (5 nM) and incubated in RPMI supplemented with 10% FCS for a further 24 h. Thereafter, cells were treated with DM and TGFβ1 (10 ng/ml) for 72 h. Following treatment; conditioned medium was removed and analysed for IGFBP-1 and PRL protein levels by ELISA and time-resolved fluorimmunoassay respectively (see below). In parallel, mRNA was prepared and analysed by Q-RT-PCR.

### Taqman Quantitative Real Time PCR (Q-RT-PCR)

RNA was extracted from cells in Tri reagent (ABgene House, Surrey, UK); RNA samples were reverse transcribed using random hexamers. Gene-Specific Primers and Probes were designed using Primer Express software (PerkinElmer/Applied Biosystems, Cheshire, UK); PRL Forward: ‘5- GCCCCGGAGGCTATCCTA-3’, dPRL Reverse, ‘5-TCAGCTCCATGCCCTCTAGAA-3’, dPRL Probe ‘5-CCAAAGCTGTAGAGATTCAGGAGCAAACCA-3’. IGFBP-1 Forward: ‘5-CACAGGAGACATCAGGAGAAGAAA-3’, IGFBP-1 Reverse: ‘5-ACACTGTCTGCTGTGATAAAATCCAT-3’, IGFBP-1 Probe: ‘5-TTCCAAATTTTACCTGCCAAACTGCAACAA-3’. Tissue Factor Forward: 5′-CAC CGA CGA GAT TGT GAA GGA-3′, Tissue Factor Reverse; 5′-CCC TGC CGG GTA GGA GAA-3′, Tissue Factor Probe: 5′-TGA AGC AGA CGT ACT TGG CAC GGG T-3′. Primers were diluted to 250 µM and probes to 50 µM in TE buffer (10 mM Tris; 1 mM EDTA in Depc H_2_O). PCR reaction mixtures contained TaqMan® Universal PCR Master Mix, No AmpErase® UNG (Applied Biosystems) (7.2 mM MgCl_2_; 1.6 mM Stratagene dNTP mix; 1.6 mM Boehringer dNTP mix; 0.05 U/µl Taq Polymerase; 2x PCR buffer and 0.06% reference dye diluted in Depc H_2_O) and specific forward and reverse primers (250 nM; Biosource, Nivelles, Belgium) and probe (50 nM; Biosource) in a final volume of 25 µl/well. Ribosomal 18S primers and probe (PE Biosystems, Warrington, UK) were added at a final concentration of at 50 nM. PCR reactions were run on ABI Prism 7900 (Applied Biosystems). Samples were measured in duplicate and mean values were used in subsequent analysis. Relative quantification was achieved using the formula 2^-ΔΔCt^, which relates the amount of cDNA of the specific amplicon to the 18S internal control and the control cDNA.

### Enzyme-Linked Immunoadsorbant Assay (ELISA)

Culture supernatants were stored at −20°C. The IGFBP-1 assay used matched antibody pairs (R&D, Abingdon, Oxford) and was conducted according to manufacturer's protocols. Non-decidualized and decidualized control samples were assayed in duplicate and the concentration of IGFBP-1 was determined by interpolation from a standard curve using known concentrations of IGFBP-1 standards. The inter-assay variation was calculated as a relative standard deviation and found to be 8.79% whilst intra-assay variation was 5.98%.

### Time-resolved fluorimmunoassay

Culture supernatants were stored at −20°C until assayed. Prolactin (PRL) release was measured by a DELFIA® Prolactin time-resolved fluoroimmunoassay kit (PerkinElmer Life Sciences). The fluoroimmunoassay was a solid phase, two-site assay based on the direct sandwich technique. The fluorescence of each sample is proportional to the concentration of PRL in the media sample and was measured on a time-resolved fluorometer, VICTOR™ 1420 Multilevel Counter (Wallac, PerkinElmer LAS (UK) Ltd, Beaconsfield, UK). The concentration of PRL was determined by interpolation from a standard curve prepared from the PRL standards. The assay was conducted according to the manufacturer's protocol. All samples from each experiment were analysed in the same assay in order to preclude inter-assay variability.

### Statistical Analysis

Prior to any statistical analysis data were tested for and shown to exhibit Gaussian distribution. Gaussian distribution was determined by applying the Shapiro-Wilk normality test to the data. Where appropriate, values were presented as means ± S.E.M. Comparison of the different parameters for the various treatment groups was determined by repeated measures analysis of variance (ANOVA). Significant differences were assigned using Kruskal-Wallis post hoc test. The criterion for significance for all tests was set at p<0.05. Specific software was used to assist in the data analysis (GraphPad Prism v4.0b for Macintosh, GraphPad Software, San Diego, USA).

## Results

### TGFβ1 down-regulates the production of decidualization markers

**Figure 1:**
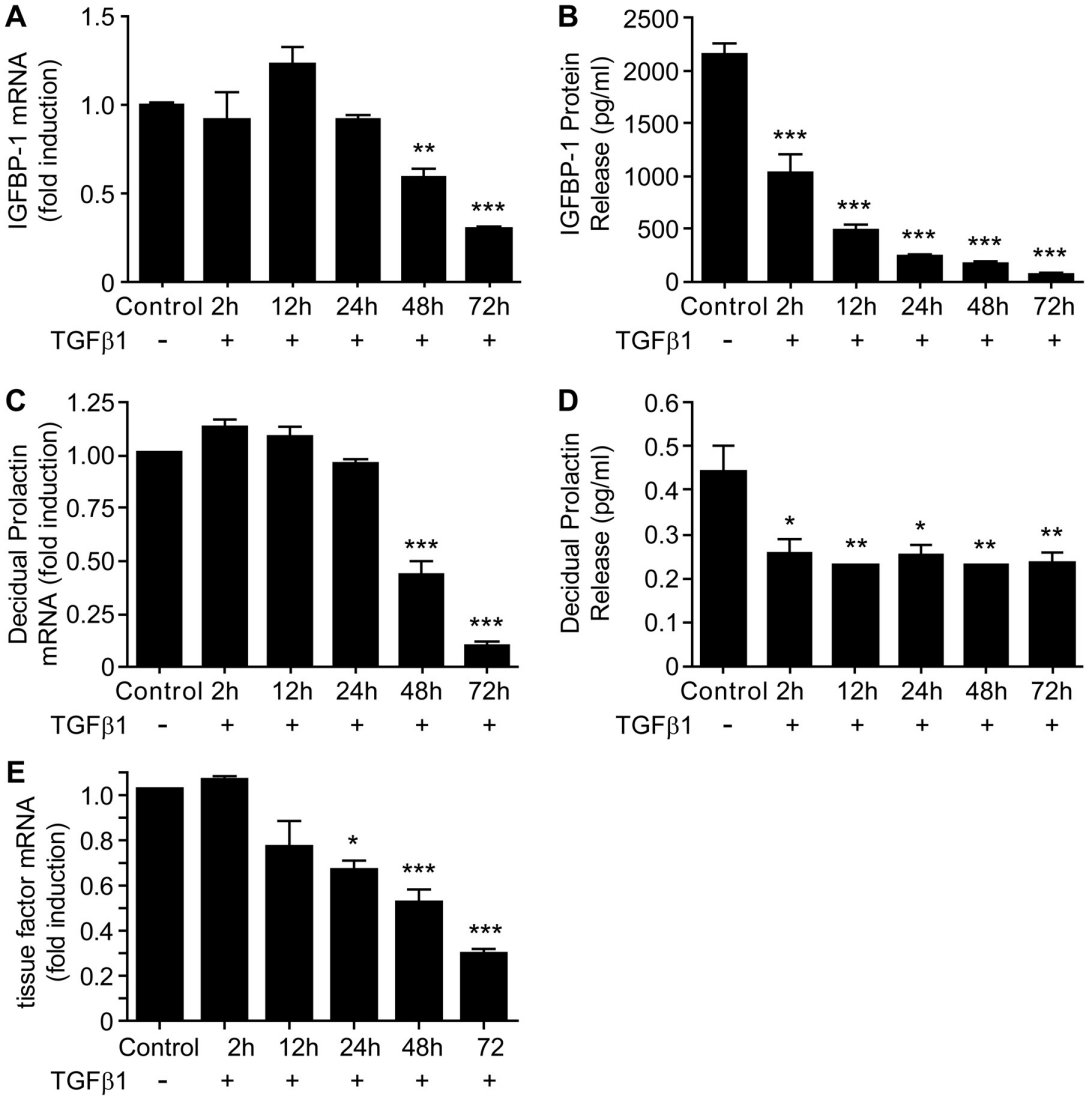
TGFβ1 inhibited expression of decidualization markers when ESC were decidualized in the presence or absence of TGFβ1 for up to 72 h. A: TGFβ1 reduced expression of mRNA IGFBP-1 in a time dependent manner, 48 h, p<0.01, 72 h, p<0.001. 1.0 =  absolute value of ΔCT  = 12. B: TGFβ1 inhibited release of IGFBP-1 protein release after only 2 h of treatment (p<0.001) and this continued to decline in a time-dependent manner (all time points p<0.001). C: TGFβ1 inhibited expression of PRL mRNA in a time dependent manner, 48 h (p<0.01), 72 h (p<0.001). 1.0 =  absolute value of ΔCT  = 11. D: TGFβ1 inhibited PRL protein release after only 2 h of treatment (p<0.05) and this was sustained for up to 72 h (12 h, p<0.01, 24 h, p<0.05, 48 h and 72 h, p<0.01). E: TGFβ1 inhibited expression of TF mRNA in a time dependent manner; 24 h (p<0.05), 48 h (p<0.001), 72 h (p<0.001). 1.0 =  absolute value of ΔCT  = 14. Data are mean ± S.E.M; * p<0.05, ** p<0.01, *** p<0.001 vs. control. n = 8 endometrial samples (in triplicate).

To determine the impact of TGFβ1 on decidualization, primary human ESC (n = 8, endometrial samples) were decidualized *in vitro* for 6 days and then further treated with DM in the presence or absence of TGFβ1 (10 ng/ml) for 72 h. Incubation of decidualized ESC with TGFβ1 down-regulated the expression of IGFBP-1 mRNA in a time-dependent manner with a significant decrease observed at 48 h (p<0.01) and 72 h (p<0.001), as compared to time-matched controls (Figure 1A). In contrast a significant decrease in the amount of IGFBP-1 protein released from the cells was detected after only 2 h incubation with TGFβ1 and the amounts declined further during the rest of the experiment (12 h, 24 h, 48 h and 72 h, Figure 1B; all p<0.001). Treatment of cells with TGFβ1 was also associated with a significant decrease in intracellular concentrations of PRL mRNA (Figure 1C) and the amount of PRL released into the culture media (Figure 1D). Notably the patterns of expression closely paralleled those observed for IGFBP with significant inhibition of mRNA levels observed at 48 h (p<0.001) and 72 h (p<0.001) but a reduction in release of PRL into the medium after only 2 h of TGFβ1 treatment (p<0.05) (Figure 1D). Treatment of decidualized ESC with TGFβ1 also reduced the amount of tissue factor mRNA, with significant suppression of mRNA levels observed at 24 h (p<0.05), 48 h (p<0.001) and 72 h (p<0.001) (Figure 1E).

### Anti-TGFβ1 neutralizing antibody negates TGFβ1 inhibition of gene expression

**Figure 2:**
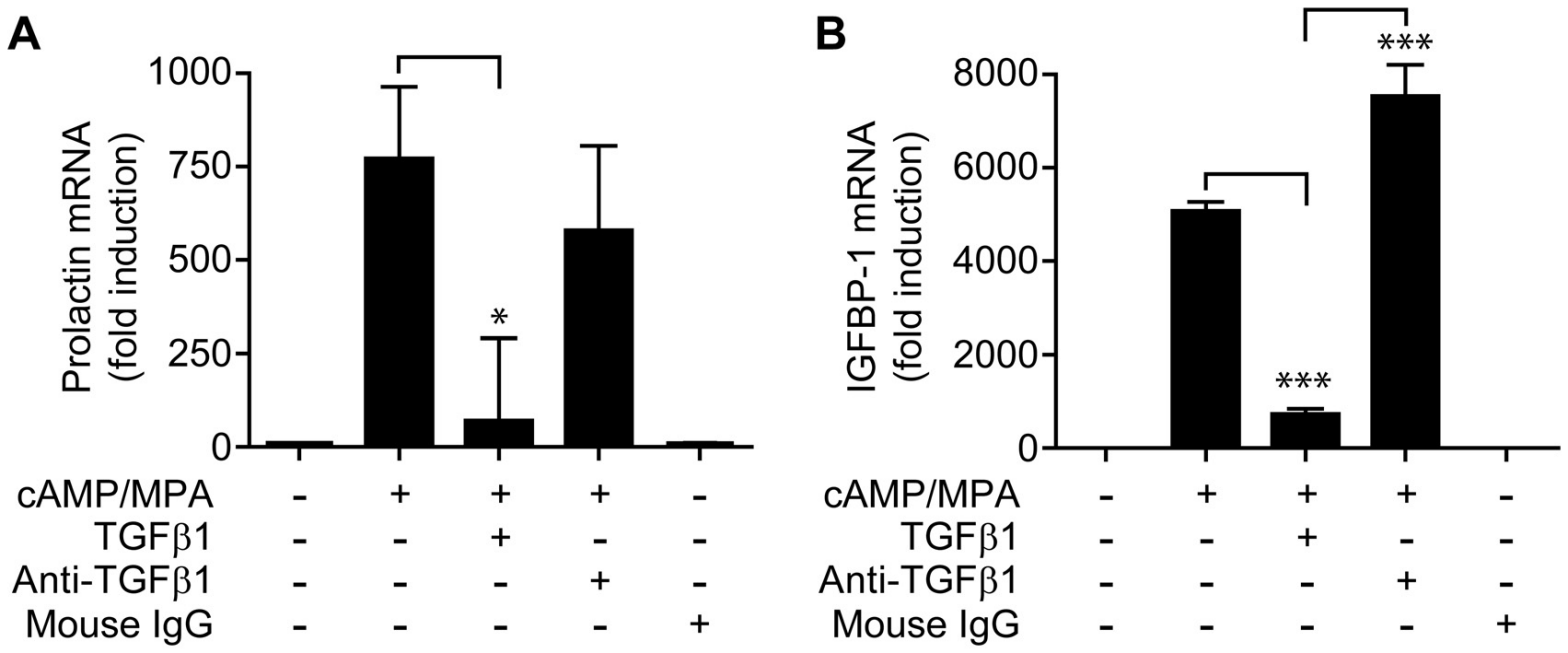
Anti-TGFβ1 antibody neutralizes endogenous TGFβ1 and potentiates the decidualization process. Cultured ESCs were decidualized *in vitro* in the prescence and absence of TGFβ1. To confirm the specificity of the TGFβ1 response anti-TGFβ1 antibody (1 µg/ml) or mouse IgG control were added, for 72 h. A: TGFβ1 inhibited expression of decidual PRL (p<0.05), addition of anti-TGFβ1 blocked this reduction. 1.0 =  absolute value of ΔCT  = 11.5. B: TGFβ1 reduced expression of IGFBP-1 mRNA (p<0.001), whilst anti-TGFβ1 antibody increased expression IGFBP-1 (p<0.001) above that of controls. 1.0 =  absolute value of ΔCT  = 12.3. Data are mean ± S.E.M; * p<0.05, *** p<0.001. n = 6 endometrial samples (in triplicate).

**Figure 3:**
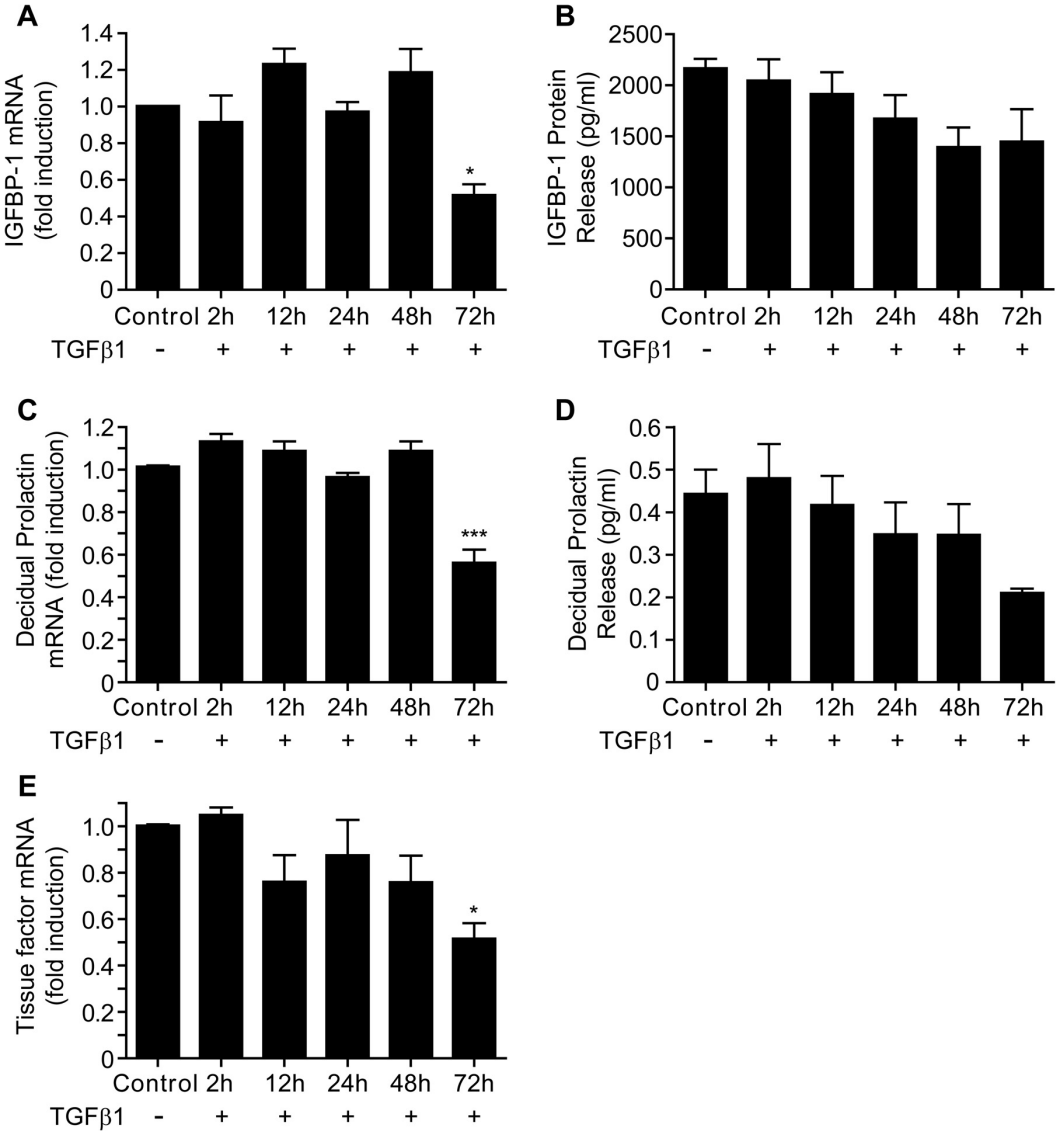
TGFβ1 suppresses expression and release of markers of decidualization by cells from 1^st^ trimester decidua. A: TGFβ1 reduced expression of IGFBP-1 mRNA after 72 h treatment. (p<0.05) 1.0 =  absolute value of ΔCT  = 12.2. B: TGFβ1 was without significant effect on protein release of IGFBP-1. C: TGFβ1 reduced expression of PRL mRNA after 72 h treatment (p<0.001). 1.0 =  absolute value of ΔCT  = 11.5. D: TGFβ1 was without significant effect on PRL protein release, although did display a trend toward inhibition. E: TGFβ1 reduced expression of TF mRNA after 72 h treatment (p<0.05). 1.0 =  absolute value of ΔCT  = 13.75. Data are mean ± S.E.M; * p<0.05, *** p<0.001 vs. no TGFβ1 treatment. n = 7 decidual samples (in triplicate).

hESC (n = 6 endometrial samples) were decidualized *in vitro* for 6 days then cultured in DM in with TGFβ1 (10 ng/ml) or an anti-TGFβ1 antibody (1 µg/ml) for a period of 72 h. As detailed above incubation with TGFβ1 significantly reduced the amount of PRL (p<0.05, Figure 2A) and IGFBP-1 (p<0.001, Figure 3B) mRNAs. We have previously demonstrated that decidualization of hESC is associated with biosynthesis of TGFβ1 [19]; in the current experiments addition of anti-TGFβ1 antibodies maintained expression of PRL mRNA (Figure 2A) and significantly potentiated expression of IGFBP-1 mRNA in comparison with ESCs treated with DM alone (p<0.001, Figure 2B).

### TGFβ1 suppresses the expression and release of PRL, IGFPB-1 and TF by cells obtained from first trimester decidua

In order to determine whether incubation with TGFβ1 had a similar impact on primary DSC to hESC incubated in vitro, cells were obtained from decidua recovered from pregnancies of <10 wks gestation. Incubation with TGFβ1 for up to 72 h (n = 7) resulted in a significant reduction in the amount of IGFBP-1 mRNA (Figure 3A, p<0.05), PRL mRNA (p<0.001, Figure 3C) and TF mRNA (p<0.05, Figure 3E) at 72 h as compared to unstimulated, time-matched controls (Figure 3). Notably in contrast to the results obtained with decidualized hESC incubation of DSC with TGFβ1 had no significant impact on the release of IGFBP-1 or PRL protein (Figure 3, B and D).

### TGFβ1 attenuates the expression and release of PRL in a SMAD 4-dependent manner

**Figure 4:**
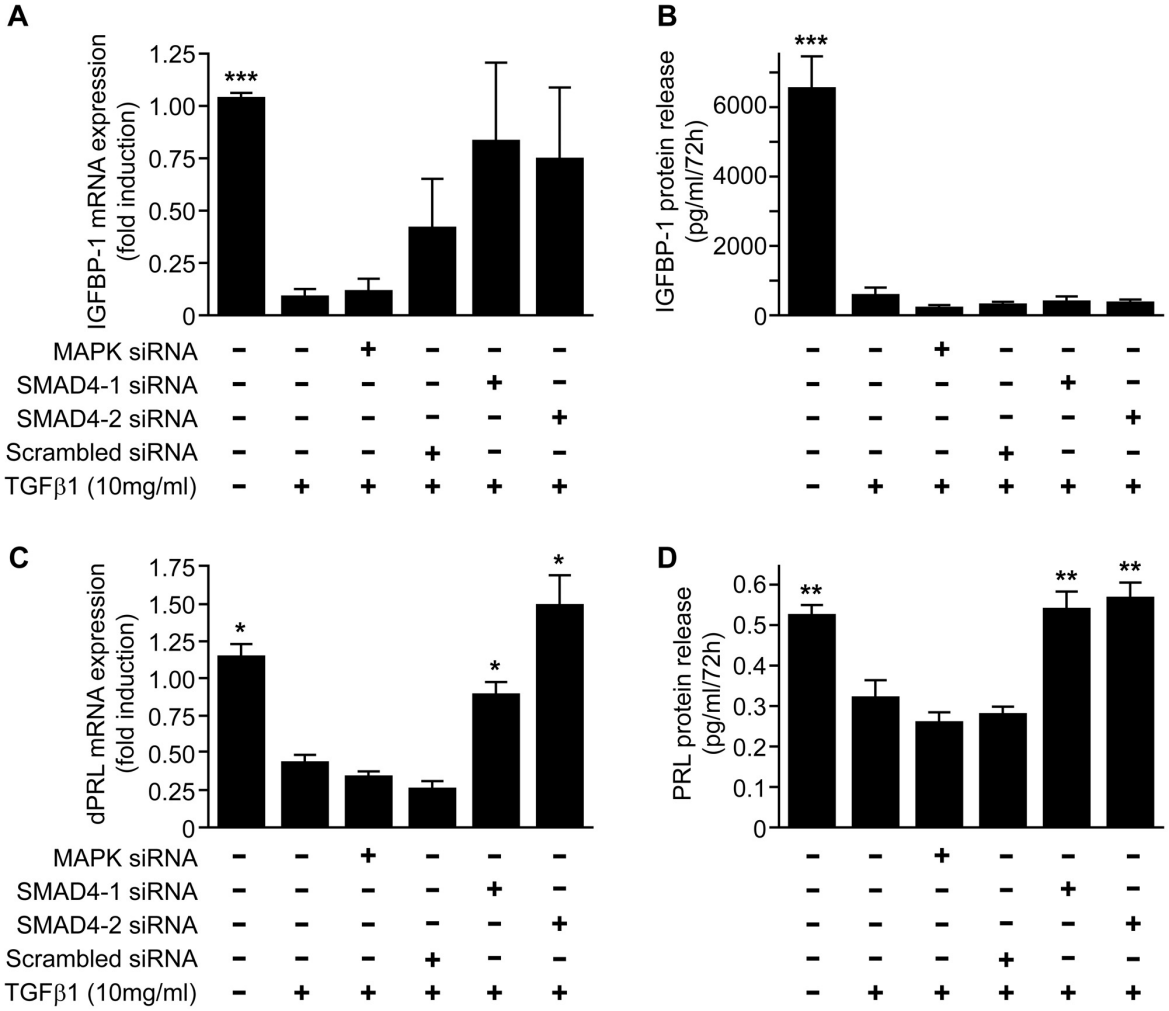
TGFβ1 attenuates expression and release of prolactin in a SMAD-dependent manner. Incubation with TGFβ1 (10 ng/ml; 72 h) significantly reduced expression of mRNAs encoding IGFBP-1 and PRL (p<0.001 IGFBP1; graph A 1.0 =  absolute value of ΔCT  = 12.2, p<0.05 PRL; graph C 1.0 =  absolute value of ΔCT  = 11) and the amount of each of these proteins recovered from culture media (p<0.001 IGFBP1, graph B, p<0.01 PRL, graph D) after incubation of decidualized ESCs for 72 h. Depletion of SMAD 4 using two independent target-specific siRNAs had no significant impact on the TGFβ1-dependent reduction in IGFBP-1 mRNA or protein (graphs A 1.0 =  absolute value of ΔCT  = 12.2, B) but reversed the reduction in PRL mRNA (p<0.001, graph C 1.0 =  absolute value of ΔCT  = 11.25) and protein (p<0.01, graph D) induced by treatment. No response was observed with cells depleted of MAPK siRNA. Data are mean ± S.E.M; * p<0.05, ** p<0.01, *** p<0.001 vs. TGFβ1 alone. n = 5 endometrial samples (in triplicate).

So as to determine whether the impact of TGFβ1 on expression and release of IGFBP-1 and PRL was mediated by the SMAD signalling pathway, cells were transfected with SMAD 4-specific siRNAs, a siRNA directed against MAPK (a pre-validated control siRNA) or RNA of an unrelated sequence. Western blotting was used to confirm >90% reduced expression of SMAD 4 (and MAPK) in cells transfected with the sequence specific siRNAs (data not shown). Targeted knockdown of SMAD 4 in decidualized ESCs using two independent siRNAs (n = 5) had no significant impact on the TGFβ1-dependent decrease in concentrations of IGFBP-1 mRNA (Figure 4A) and release of IGFBP-1 protein remained significantly depressed in all cells treated with TGFβ1 regardless of the addition of any of the siRNAs (Figure 4B). In contrast, targeted knockdown of SMAD 4 prevented the TGFβ1-dependent decrease in expression of PRL mRNA (Figure 4C) and the amount of PRL released (Figure 4D) remained at control levels; the amount of PRL released by cells transfected with an siRNA directed against MAPK was similar to that released by cells incubated with TGFβ1 alone.

## Discussion

In the present study we have demonstrated that TGFβ1 reduces the expression and secretion of PRL, IGFBP-1, and TF by human ESCs decidualized *in vitro*, as well as primary DSCs obtained from 1^st^ trimester decidua. Notably the latter appeared more refractory to the treatment. Targeted knockdown of SMAD 4, the protein which translocates phosphorylated SMAD members to the nucleus mediating the transcriptional downstream biological actions of TGFβ1, [18] revealed that the impact of TGFβ1 on expression and release of IGFBP1was SMAD independent. In contrast inhibition of PRL protein release was SMAD-dependent demonstrating that TGFβ1 can act via more than one signalling pathway in this cell type.

Previous studies have reported that TGFβ1 can alter expression of decidual proteins although impacts on endometrial decidualization have been inconsistent. To our knowledge the current study reports the first data directly comparing the response to TGFβ1 in cells decidualized *in vitro* with primary cells recovered from decidua i.e. those exposed to the presence of a blastocyst. Primary ESCs, obtained from non-pregnant endometrium and decidualized *in vitro*, are considered a model for cells that decidualize during the non-pregnant menstrual cycle. In primary ESCs we demonstrated incubation of cells with TGFβ1 reduced both the concentrations of IGFBP-1 and PRL mRNAs as well as the amounts of these proteins secreted into the culture media. The findings in the current study are in agreement with a number of studies reporting a marked inhibitory effect of TGFβ1 on basal and stimulated PRL secretion, mRNA levels and *de novo* PRL synthesis in rat anterior pituitary cells [25], decidual cells from 1^st^ trimester [26] and term pregnancy [27]. However in contrast to the current findings, it has been reported that TGFβ1 can *potentiate* the decidualization process in ESCs with increased production of PRL independent of the presence of progesterone [21], [22]. With a further study reporting a TGFβ1-dependent increase in expression of PRL in ESCs [28] although these cells were not exposed to a decidualization stimulus. One limitation to our study is that all the decidual markers we examined are also regulated by progesterone. As we have cultured all our cells in the presence of MPA (decidualization stimulus) we are unable to reject the possibility that augmentation of the decidual markers is occurring as an indirect consequence of TGFβ1 mediated suppression of PR expression [19]. Interestingly, we detected a very rapid reduction in protein release for both IGFBP-1 and PRL in ESCs that preceded any reduction in total concentrations of the mRNAs. This would suggest that TGFβ1 might also be repressing translation/export of proteins or could be modulating expression of tissue-specific microRNAs (miRNAs), short nucleotide sequences involved in post-transcriptional gene regulation that have been implicated in endometrial function [29], [30]. However, no direct association between TGFβ1 and recently identified menstruation-specific miRNAs has been identified [31] and these suggestions therefore remain speculative. The impact of TGFβ1 on functional activity and differentiation of ESC during the normal cycle may also extend beyond the impact on decidualization as studies using primary ESC reporting that TGFβ1 down-regulates PR expression [19] and inhibits cell proliferation and migration [32]. Together with evidence detailing that *in vivo* TGFβ1 expression is increased at menstruation [33], and TGFβ1 can induce contraction of decidualized stromal cells [34] it has been proposed that TGFβ1 may play a role in the onset of menstruation in normal cycling endometrium. Furthermore, aberrant expression of TGFβ1 may contribute to menstrual disorders, such as heavy menstrual bleeding and painful menstruation, by modifying local haemostatic mechanisms (reviewed in [35]).

To determine if decidualized stromal cells would respond to TGFβ1 in the same manner as primary ESCs that were decidualized in vitro, we isolated stromal cells from first trimester decidua. Although, the present studies have demonstrated that TGFβ1 markedly inhibits the expression of PRL, IGFBP-1, and TF mRNAs in DSCs this inhibition was delayed by at least 24 h when compared to the response observed in ESC. Furthermore, this inhibitory effect at the level of mRNA was not reflected by a reduction in mature protein secretion of PRL and IGFBP-1 by DSC, implying that decidualization *in vivo*, confers some resistance to the actions of TGFβ1. The findings in the current study are in agreement with studies reporting that TGFβ1 inhibited both IGFBP-1 and PRL production in a time-dependent manner in decidual cells from 1^st^ trimester [26], [36] and term pregnancy [27]. However, the effect of TGFβ1 on PR expression in DSCs remains unknown making it difficult to interpret the results in terms of direct effects of TGFβ1 on DSCs as opposed to an indirect effect via suppression of PR [19]. It may be that the presence of a blastocyst and increasing concentrations of hCG in the first trimester of pregnancy evokes an increase in cellular protection against potentially harmful cytokines and growth factors. Alternatively, the role of TGFβ1 in pregnant endometrium could differ from that in non-pregnant, pre-menstrual endometrium. This view is supported by data reporting high expression of TGFβ1 in first trimester decidua without any detrimental effect on pregnancy [37], [38], [39]. Aberrant increases in active TGFβ1 during early pregnancy may be detrimental as a consequence of inadequate decidualization of the endometrium.

To determine if TGFβ1 was conferring its actions via its canonical signalling cascade we interrogated the common mediator of all SMAD signal transduction, SMAD 4 [40]. We have demonstrated via knockdown of SMAD 4 that TGFβ1-induced suppression of IGFBP-1 is not SMAD-dependent and these data suggest that TGFβ1 is mediating its effects via an alternative pathway or an indirect mechanism, as has been suggested previously [41]. This may include the involvement of Wnt signalling pathways as previous reports have shown that progesterone-dependent changes in expression of the Wnt antagonist DKK parallel changes in secretion of IGFBP-1 protein [42]. This report was complemented by our own study demonstrating that TGFβ1 inhibits expression of DKK-1 mRNA in a SMAD-independent manner [19]. In contrast TGFβ1-specific down regulation of expression of PRL was SMAD-dependent and the impact of TGFβ1 was reversed in cells transfected with SMAD-4 specific si-RNAs. This finding would be consistent with previous reports demonstrating that activin-dependent inhibition of expression of PRL in the pituitary is mediated by the SMAD signalling pathway [43]. Other studies reporting conflicting results to our own have also demonstrated a role for SMAD signalling in propagating TGFβ1 actions [22], [28], with the authors claiming that both ERK and SMAD dependent signalling may play a role in the TGFβ1-dependent increase in expression of PRL in ESC [28]. However, in contrast, the impact of TGFβ1 in our decidualized cells appeared to be independent of expression of MAPK. It is likely that TGFβ1 may be evoking responses in genes that are not normally associated with decidualization e.g induction of smooth muscle actin α (Kane *et al*, unpublished observations); however genome-wide transcriptional profiling is beyond the scope of this research.

In summary, the findings presented in the current study have demonstrated that TGFβ1 is capable of suppressing expression and secretion of decidualization marker proteins via both SMAD-dependent and independent mechanisms. Our findings support the hypothesis that local TGFβ1 signalling may coordinate de-differentiation of endometrial stromal compartment and tissue remodelling associated with menstruation, but raise the possibility that this factor may play a different role in the pregnant endometrium.
